# Use of a voluntary testing program to study the spatial epidemiology of Johne’s disease affecting dairy herds in Minnesota: a cross sectional study

**DOI:** 10.1186/s12917-019-2155-7

**Published:** 2019-12-02

**Authors:** K. S. T. Kanankege, G. Machado, L. Zhang, B. Dokkebakken, V. Schumann, S. J. Wells, A. M. Perez, J. Alvarez

**Affiliations:** 1 Department of Population Medicine, College of VeterinaryMedicine, University of Minnesota, 1365, Gortner Avenue, St. Paul, MN 55108 USA; 2 Department of Population Health and Pathobiology, College of Veterinary Medicine, North Carolina State University, Raleigh, USA; 3 Division of Biostatistics, School of Public Health, University of Minnesota, Minneapolis, USA; 4 Minnesota Dairy Herd Improvement Association, Buffalo, USA; 5 Centro de Vigilancia Sanitaria Veterinaria (VISAVET), Departamento de Sanidad Animal, Facultad de Veterinaria, Universidad Complutense, Madrid, Spain

**Keywords:** Paratuberculosis, Milk ELISA, Spatial epidemiology, CAR model, Regression, Passive surveillance

## Abstract

**Background:**

One of the key steps in the management of chronic diseases in animals including Johne’s disease (JD), caused by *Mycobacterium avium subsp. paratuberculosis* (MAP), is the ability to track disease incidence over space and time. JD surveillance in the U.S. dairy cattle is challenging due to lack of regulatory requirements, imperfect diagnostic tests, and associated expenses, including time and labor. An alternative approach is to use voluntary testing programs. Here, data from a voluntary JD testing program, conducted by the Minnesota Dairy Herd Improvement Association, were used to: a) explore whether such a program provides representative information on JD-prevalence in Minnesota dairy herds, b) estimate JD distribution, and, c) identify herd and environmental factors associated with finding JD-positive cows. Milk samples (*n* = 70,809) collected from 54,652 unique cows from 600 Minnesota dairy herds between November 2014 and April 2017 were tested using a MAP antibody ELISA. Participant representativeness was assessed by comparing the number of JD-tested herds with the number of herds required to estimate the true disease prevalence per county based on official statistics from the National Agricultural Statistical Services. Multivariable logistic regression models, with and without spatial dependence between observations, were then used to investigate the association between herd status to JD (positive/negative), as indicated by milk ELISA results, and available covariates at the herd level.

**Results:**

Within the study population, at least one test-positive cow was found in 414 of 600 (69%) herds. Results indicated that large herds that test frequently and herds located in loamy or silt soils are more likely to have at least one MAP test-positive cow. After adjusting for herd size, testing frequency, and soil type, there was no spatial dependence in JD risk between neighboring dairies within 5 to 20 km. Furthermore, the importance of collecting data on herd management, feed, and biosecurity for insightful interpretations was recognized. The study suggested that, although limited, the voluntary testing database may support monitoring JD status.

**Conclusions:**

Results presented here help elucidate the spatial characteristics of JD in Minnesota and the study may ultimately contribute to the design and implementation of surveillance programs for the disease.

## Background

Johne’s disease (JD) is a chronic, debilitating enteritis that affects ruminants and is caused by infection with *Mycobacterium avium subsp. paratuberculosis* (MAP) [1]. Financial losses due to JD in the U.S. dairy industry have been estimated to range between $200 to 250 million USD annually [2]. Reduced milk production and quality due to reduced fat and protein content, increased premature mortality, weight loss, early culling, costs of testing and control, and reduced slaughter value are among the negative impacts of JD [3–6]. In addition, although not confirmed, the potential link between MAP and the development of Crohn’s disease in humans further increases the hypothetical importance of JD [7]. A recent review highlighted that the global prevalence of JD is underestimated and setting objectives for surveillance and control measures is much needed [8].

The management and control of a chronic disease such as JD in a proactive and organized manner is challenging in the U.S. due to lack of regulatory requirements for testing [9], imperfect diagnostic tests [10], long-term survival of the pathogen outside the host [11], multiple routes of transmission, and the cost and labor necessary for long-term disease tracking [12].

JD is widespread in the U. S, and herd prevalence has been estimated as 60.7% in Midwestern U.S. dairies [13] and 91.1% nationally [14]. However, JD control is voluntary in the US and therefore testing for JD is not mandatory [9], which limits the availability of data and resources to monitor the disease. Studies suggest that limited adoption and compliance with JD testing and control strategies in dairy farms is a result of a) the chronic nature of the disease progression, therefore, the absence of the “cues-to-action” [15], b) the farmers’ perception of the limited cost-effectiveness of the herd control measures [16], and c) not perceiving JD as a “hot topic” during communications with other farmers and veterinarians [16].

Due to the lack of official disease monitoring, a common alternative for evaluating the epidemiological status of JD in a region is the use of data from voluntary testing programs, such as those collected by the Minnesota Dairy Herd Improvement Association (MNDHIA). Minnesota, a Midwestern state of the U.S., has nearly 460,000 dairy cattle and is among the top ten dairy states, 6th in terms of milk cow numbers and 8th in dairy herd numbers, per 2016 statistics available from the National Agricultural Statistics Services (NASS) [17, 18]. A proportion of Minnesota dairy farms utilize the services of MNDHIA, a member of the National Dairy Herd Improvement Association who provide a testing and production recordkeeping service to U.S. dairy farms (http://www.dhia.org/members.asp). However, it is unknown if data collected by the MNDHIA is useful as a passive surveillance tool to monitor JD in Minnesota.

The objectives of the study here were to a) test whether the voluntary JD testing program conducted by the MNDHIA can provide representative information on the prevalence of JD in dairy herds in Minnesota, b) estimate the JD distribution in Minnesota using data from the MNDHIA voluntary JD testing program, and c) identify possible herd and environmental variables associated with increased risk of having JD milk ELISA test-positive cows, using the available data. We hypothesized that results from the voluntary JD program might be used to evaluate JD status in an area and inform management decisions made by the testing agencies, veterinarians, and dairy producers. In evaluating the use of the MNDHIA database as a passive surveillance tool, our overarching objective was to generate evidence that could influence management decisions by recognizing modifiable factors to reduce the JD risk at the individual, herd, and regional levels. Results could therefore be useful in the design and implementation of surveillance programs for the U.S. dairy industry.

## Results

### Spatial representativeness

**Fig. 1:**
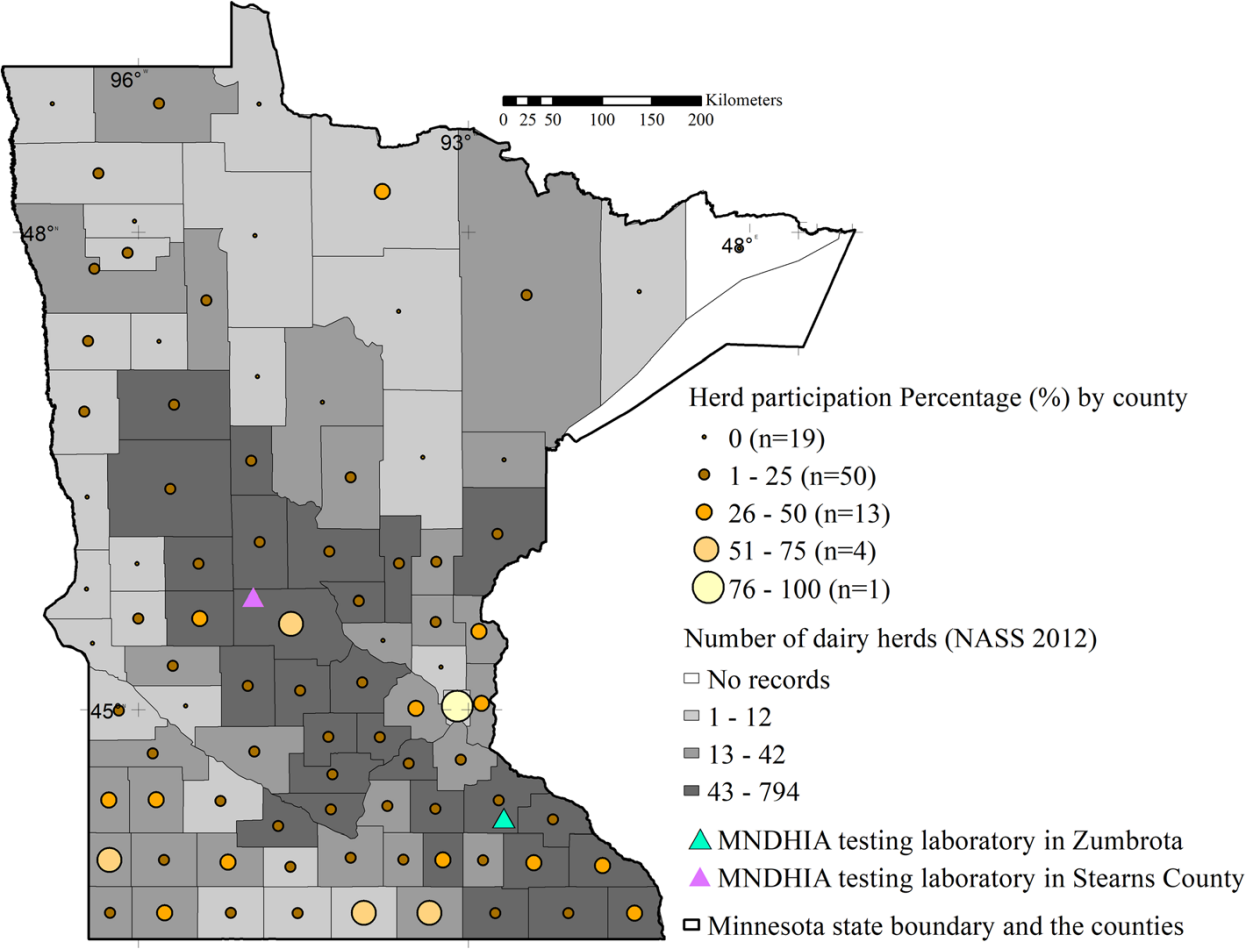
The participation of study herds as a percentage of the ideal sample size, by county, is summarised with the graduated symbols. The Minnesota Dairy Herd Improvement Association testing laboratories are illustrated with triangles. The background colors in grey indicates the number of dairy herds in each county, based on 2012 census of the National Agricultural Statistics Service [18]. Map depicted here was generated as part of the current study

During the 2.5-year study period, 600/4746 (13%) dairy herds in Minnesota tested at least once for JD at MNDHIA laboratories, representing 18.7% (600/3210) of the licensed dairy herds in Minnesota with permits to ship milk for human consumption [17]. Figure 1 depicts the number of MNDHIA sampled dairy herds by county, the minimum required number of herds to estimate a true JD prevalence, and the number of dairy herds listed in the 2012 NASS Census of Agriculture (by terciles). We observed that the distribution of the MNDHIA participants included in this study mirrors the pattern of the milk cow herds included in the USDA NASS 2012 report (Fig. 1). Nevertheless, the minimum sample size required to estimate disease prevalence was not attained in any Minnesota county except Ramsey (where both the appropriate and observed sample sizes were 1).

### Descriptive statistics and spatial pattern recognition

**Fig. 2:**
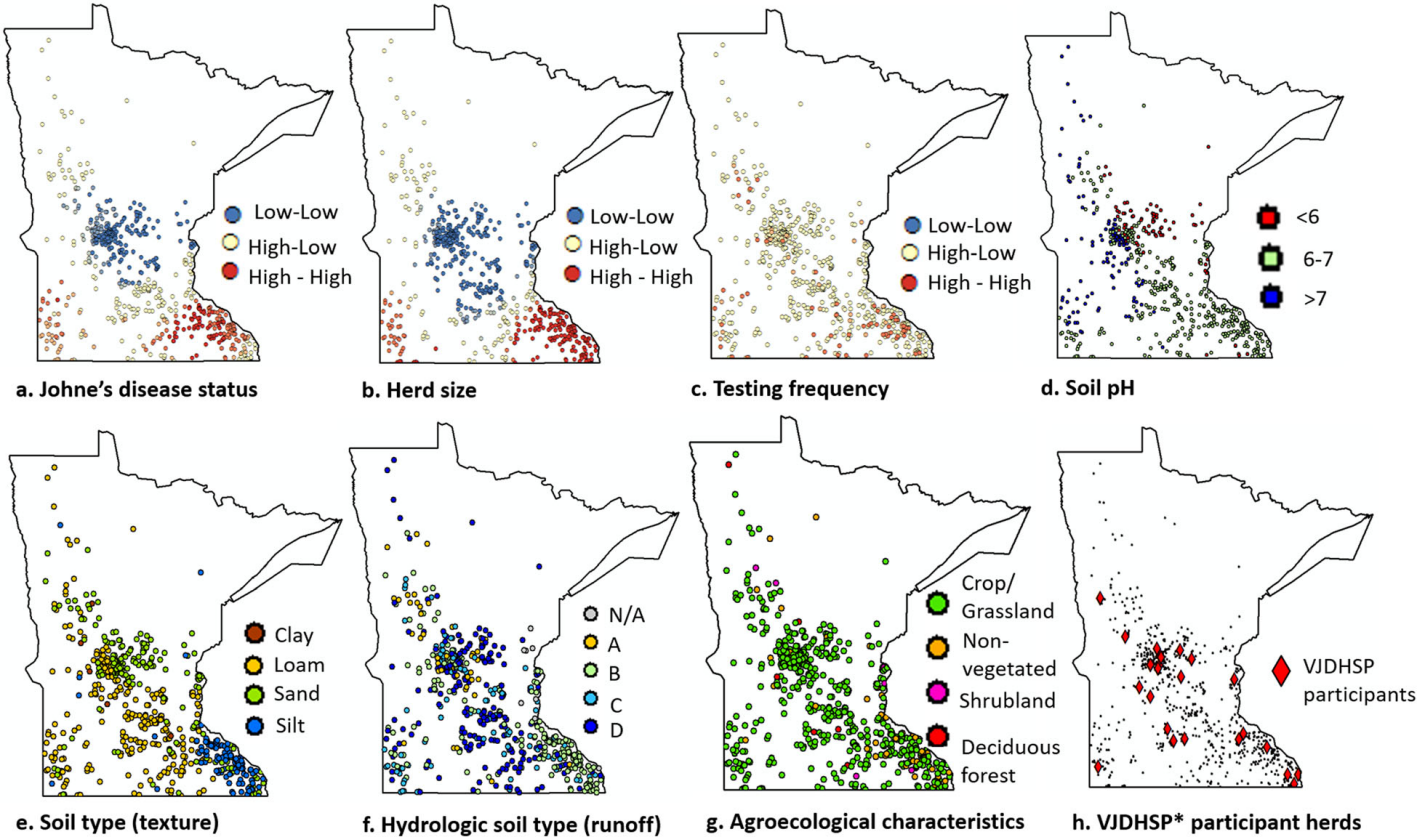
Spatial patterns of Johne’s disease status and the associated covariates. Points represent the location of study herds (*n* = 600). Panels (**a**, **b**, and **c**) illustrates the results of the Getis Ord Gi* local test where herds with high value of the variable next to herds with high values of the variable are represented in red (high-high clusters), herds with low value of the variable next to herds with low values of the variable represented in blue (low-low clusters), and no-matching pairs in yellow (high-low, or low-high values) [19, 20]. Covariates depicted in panels d though h include: (**d**) soil pH; (**e**) soil type/texture; (**f**) hydrologic soil type (i.e. runoff); (**g**) Agro-ecological characteristics; and (**h**) participants of the Voluntary Johne’s Herd Status Program for Cattle program (VJDHSP*). Maps depicted here were generated as part of the current study

The apparent herd-level prevalence of MAP, based on having 1+ cows with a positive milk ELISA, was 69% (414/600). The MNDHIA herds in this study included both small (< 100 cows, *n* = 332) and large (≥ 100 cows, *n* = 268) dairies. Figure 2 illustrates the Getis ord Gi* local test results for JD status and the covariates in the geographical space According to the Getis Ord Gi* analysis, we concluded that a certain risk of JD infection was present in herds throughout Minnesota, although herds in the southeastern corner were more likely to be JD milk ELISA test-positive compared to the herds in the northcentral region (Fig. 2: Panel a). The herd size of the study population showed a similar pattern where larger herds were located in the southeastern corner of the state, while smaller herds were in the northcentral region (Fig. 2: Panel b). We observed no spatial pattern in the testing frequency (Fig. 2: Panel c). Spatial patterns were observed for many of the remaining covariates: soil pH, soil type (texture), soil hydrologic characteristics (runoff potential), and agroecological characteristics (Fig. 2). The soil pH in Western Minnesota contained predominantly alkaline soils. However, the soil type (texture), hydrologic soil types, and agroecological characteristics demonstrated intricate patterns of spatial distribution. According to the Cramer’s V, none of the covariates were strongly associated with each other.

### Classification of JD status

Based on the appropriate number, i.e. minimum sample size per herd (Table 2) criterion, we observed 437/600 (72.83%) herds that had tested an appropriate number of cows during the study period. Of those 437 herds, 186 herds (31%) had no test-positive cows. As mentioned above, all other herds had at least one positive cow (414/600; 69%). Although the study presented here considered the entire study period, the suggested minimum sample sizes listed in Table 2 are the number of cows to be tested at each testing cycle.

### Regression results

Herd size, testing frequency, and soil type (texture) were retained in the final multivariable model (Table 3). Herd turnover rates were available only from 454/600 (76%) herds and the association between JD status and herd turnover rate was not statistically significant in the univariate analysis (Table 3), therefore, excluded from the final multivariable model. The Moran’s I and Getis Ord Gi* statistics indicated that there was no spatial autocorrelation in the regression residuals (*p*-value > 0.05). Similarly, results of the fitted CAR model suggested that the spatial dependence for dairy herds located between 5 and 20 km was not significant after adjusting for the covariates, and its AIC value was higher than that of the model without the spatial component (Additional file 2: Table S2). The spatial correlation parameter of the CAR model (λ) was not significantly different from zero for any of the distance thresholds considered (Table 3).

## Discussion

This study suggests the use of an existing voluntary testing program as a passive surveillance system to track JD in Minnesota. We demonstrated that the MNDHIA voluntary testing program may be a useful source to investigate the JD status of Minnesota dairies, with program participants in 2014–2017 representing 13% of the dairy herds across Minnesota and coming from areas with both high and low density of dairy farms. Even though county-level JD herd prevalence could not be reliably estimated because the required sample sizes were not achieved in most counties, we were able to estimate the herd-level JD status for the study area as 69%, as 414 of the 600 herds had at least one cow tested-positive for JD milk ELISA. As per the epidemiological factor analysis, the most important epidemiological factors contributing to the JD status of a herd were herd size, testing frequency, and soil type, i.e. texture. We did not observe spatial dependence of the residuals of the regression model indicating that the observation of similar characteristics in JD status in the participant dairy herds were explained by the three covariates, namely, herd size, testing frequency, and soil type/texture. These results will be used to inform the potential use of the database as a surveillance tool and to suggest improvements in JD testing program conducted by MNDHIA.

JD positive herds were distributed throughout Minnesota, although herds in the southern region were more likely to be JD milk ELISA test-positive compared to herds in the north-central region (Fig. 2: Panel a). Interestingly, the herd size of the study population showed a comparable pattern, with larger herds more likely in the southern region compared to smaller herds in the north-central region of the state (Fig. 2: Panel b). A similar observation where herd size was not evenly distributed in space, with larger herds being preferentially distributed in certain areas, was also found among participants in a Danish JD control program [21].

The herds located on silt, loam, or clay soils were more likely to be JD positive compared to the herds located on sandy soils. This observation contradicts early studies in the Midwest [22], which found that high silt content was associated with reduced detection of JD. However, studies by Dhand et al. [23] and Salgado et al. [24] described the potentially higher likelihood of detecting JD on loamy and clay soils, respectively. This survival of MAP on loamy soil was experimentally observed by Salgado et al. [24], which concluded that MAP tends to migrate slower through loamy soils compared to sandy soils and thus loamy soils may have MAP contaminated upper soil layers and pasture. Furthermore, according to Salgado et al. [24], in addition to soil type, the amount of rainfall and the soil pH also play an important role in the fate of MAP in the environment. Soil hydrologic characteristics and pH were not included in the final model, but it is worthwhile to acknowledge that soil texture, pH, and hydrologic characteristics are interconnected [25] and further analysis is needed to understand the association of soil features and the persistence of MAP in manure contaminated environments [26].

Some of the limitations associated with this study include the limited generalizability of the results because MNDHIA data represents a purposive, non-random proportion of dairy herds in Minnesota. Moreover, the lack of information on herd characteristics other than herd size and testing frequency is another limitation that should be considered when interpreting the results. A more insightful interpretation requires herd management details including maternity pen management, which cows were chosen for testing for JD (high-risk cows or cows that were being sold), and the management decisions towards JD positive cows [27]. Although the long-term survival of MAP in loamy or clay soils was suggested in other studies [23], we acknowledge that additional information on the access of cattle to pastures would be necessary to establish a causal relationship between soil type and MAP exposure. Other potential routes of exposure through forages originated from other locations or grazing on pasture lands in different geographical areas such as rented pasture lands located elsewhere would certainly affect this interpretation. When data arise from imperfect surveillance systems, the interpretation of results must be done with caution because the covariates can be related either to the occurrence of the disease or to the efficiency of the data collection system [28].

Although MNDHIA does not currently offer a control program, through this study we recognized opportunities to improve the MNDHIA database to be used as a passive surveillance tool. These opportunities include: the determination of the number of herds to be sampled to establish the prevalence of JD in Minnesota, the determination of the number of animals to be sampled from each herd to ensure a reliable evaluation of its JD status (positive/negative), ensuring that farmers provide accurate farm location information, and regularly collecting herd-level information on other relevant factors such as biosecurity measures to facilitate a better assessment of JD status/risk. Moreover, having the disease and underlying factors collected frequently over the time would facilitate conducting spatiotemporal analysis and enable facilitate making temporal confidence of “disease free zones” instead of static estimates generated through a cross-sectional analysis.

A JD surveillance program would be costly to establish and a voluntary testing database could be preferred for monitoring endemic pathogens causing chronic diseases like MAP. Thus, the strength of using MNDHIA data as a source is that there are 16National Dairy Herd Improvement (DHI) Association laboratories across the U.S. (http://www.dhia.org/members.asp) and their network already acts as a record keeping system for dairy farms. Having a system to evaluate the JD status in a region would benefit the dairy industry in multiple ways, such as recognizing the differences between participant and non-participant dairy farms in the voluntary testing programs, understanding underlying risk factors and covariates in the neighborhoods, and eventually recognizing disease-free areas [6]. Moreover, records available from DHI and the Council of Dairy Cattle Breeding (https://queries.uscdcb.com/) together would have been a useful system to trace the movements/transfers or termination of individual cows, which could facilitate further investigations of how cattle movement play a role in the JD transmission. However, it is important to recognize that the choice to test for JD may not necessarily overlap temporally to make a causal association between cow transfers and JD.

Participation in voluntary testing and control programs varies due to multiple factors such as: a) farmers’ belief in the importance of JD [16], b) farmers’ belief in control and prevention strategies including the investment of time and resources [16], and c) availability of the testing facilities and trained personnel to conduct testing at convenience [29, 30]. An examination of the reasons why dairy farmers choose to test, or not, for JD status exceeded the scope of this study, although such assessment may be of interest in assessing the value of voluntary testing and control programs. This study elucidates the evaluation of JD at herd and regional level using available data. The individual-level data analysis of the same dataset was presented elsewhere [31].

## Conclusion

In summary, results reported here suggest that a routinely generated database obtained from a voluntary testing program can be used as a passive surveillance tool to monitor the infection status and epidemiological determinants of JD in a region. However, because the risk of introduction may always be present, successful prevention and control of JD depends on ongoing willingness to continue funding surveillance and research on the disease by both animal health authorities and the support from the community and stakeholders of livestock industry [32, 33].

## Methods

### Data

Data from the voluntary JD testing program conducted by the MNDHIA were collected through a 2.5-year period, between November 1st, 2014 and April 30th, 2017. Although there were records from 723 JD testing herds, 123 herds were excluded from the study (Additional file 1: Figure S1). The reasons for exclusions were: herds located outside Minnesota; herds without location information, or herds that did not test the minimum number of cows required for the study, as explained below.

Milk samples (*n* = 70,809) collected from 54,652 unique cows from 600 Minnesota dairy herds were included in the study. Samples were analyzed using the IDEXX MAP Enzyme-Linked Immunosorbent Assay (ELISA) (IDEXX Laboratories, Inc., Maine, USA) for detection of antibodies against MAP in milk. Some herds (208/600; 35%) were tested only once during the 2.5-year study period. The median number of times a herd was tested was 2 (interquartile range between 1 and 11).

To identify relevant herd and environmental factors associated with JD risk at the herd level, the scientific databases ‘Web of Science’ and ‘PubMed’ were queried to find publications using the following search string: ‘Johne’s disease,’ AND ‘*Mycobacterium avium subsp. paratuberculosis*,’ AND ‘dairy cattle,’ AND ‘risk factors’. Reviews published in peer-reviewed journals in English language were selected, and reviews on human Crohn’s disease were excluded. A total of seven reviews published between 2001 and 2017 on JD [32, 34–39] were used to identify the most commonly recognized JD risk factors in North American dairy farms. Reviews were then examined for identification of relevant risk factors for North America, and primary articles cited were used for identification of the variables to be considered in this study (Table 1). Due to unavailability of relevant data in our dataset, the following herd features were excluded from analysis: manure management, immediate culling of JD positive cows, management of the maternity pens and calves, and maintaining closed herd or purchasing animals from farms with improved management practices to control JD [12].

**Table 1:** Herd demographic factors and environmental factors associated with JD in North American dairy cattle, according to the published literature

	Covariates	Reference
1	Herd size	[38–40]
2	Testing frequency	[29]
3	Soil type (texture)	[22]
4	Soil pH	[41, 42]
5	Soil hydrologic characteristics i.e. run-off potential	[22, 43]
6	Agroecological zone (Montane/Boreal forest/Grassland/Parkland)	[39]
7	Participating the USDA Voluntary Johne’s Disease Herd Status Program i.e. VJDHSP (*n* = 24)	[9, 30]
8	Spatial dependence: Presence of neighboring dairy farms	[44]

**Table 2:** Minimum sample sizes required to estimate freedom from Johne’s Disease (JD) at the herd level, using an imperfect test and adjusting for a finite population calculated using the AusVet EpiTool Epidemiological calculator (URL: http://epitools.ausvet.com.au)

Herd size (number of cows)	Sample size^a^
5–20	N/A (Recommendation: Same as herd size)
21–25	20
26–40	24
41–55	25
56–65	26
66–80	27
81–95	28
96–100	29
101–250	30
> 251	31

^a^Assumptions: a) design prevalence, i.e. expected within herd prevalence =10%, b) sensitivity of the diagnostic test of 52%, and c) expected number of test infected cows in a herd is a product of herd size and the design prevalence

**Table 3:** Odds ratios, coefficients, and *p*-values of the association between epidemiological factors and herd-level Johne’s disease status, based on ELISA assays performed on individual milk samples in 600 herds in Minnesota

Covariates	*N*	Univariable analysis		Multivariable analysis			
		Odds ratio (CI_95%_)	*p*-value^*^	β	S.E.	Odds ratio (CI_95%_)	*p*-value^*^
Herd size			< 0.001				< 0.001
1 to ≤195	476	Ref	–	–	–	–	–
196 to ≤553	100	1.67(1.39–2.02)	< 0.001	1.58	0.43	4.84(2.20–12.27)	< 0.001
554 to 1929	24	13.74(2.86–246.87)	0.011	1.54	1.08	4.67(0.83–88.29)	0.153
Testing frequency			< 0.001				< 0.001
1	208	Ref	–	–	–	–	–
> 1 to ≤5	208	3.04(2.03–4.57)	< 0.001	1.03	0.21	2.81(1.86–4.29)	< 0.001
6 to ≤20	80	13.15(6.16–32.64)	< 0.001	2.48	0.43	11.85(5.44–29.82)	< 0.001
21 to 30	104	64.30(19.64–396.39)	< 0.001	3.77	0.73	43.34(13.00–269.33)	< 0.001
Soil type (texture)			< 0.001				0.017
Sand	189	Ref	–	–	–	–	–
Silt	115	2.87(1.69–5.01)	< 0.001	0.63	0.32	1.88(1.01–3.54)	0.047
Loam	290	1.79(1.21–2.63)	0.003	0.63	0.22	1.88(1.21–2.93)	0.005
Clay	6	3.59(0.56–69.51)	0.25	1.62	1.15	5.04(0.68–102.54)	0.162
Herd turnover rate			0.164				
18 to 37	192	Ref					
38 to 50	236	1.02(0.93–1.11)	0.698				
51 to 78	26	0.79(0.66–0.95)	0.014				
Soil pH			0.004				
< 6.0	44	Ref	–				
6.0 to 7.0	467	2.15(1.14–4.03)	0.02				
> 7.0	89	2.21(1.05–4.70)	0.037				
Hydrologic soil type			0.87				
A	57	Ref					
B	205	1.23(0.64–2.29)	0.527				
C	119	0.95(0.47–1.85)	0.874				
D	211	0.95(0.49–1.76)	0.872				
Participation in VJDHSP^a^			0.84				
Non-participants	576	Ref	–				
Participants	24	1.09(0.46–2.87)	0.84				
Agroecological zone			0.57				
Crop/Grassland	534	Ref	–				
Non-vegetated land	52	0.85(0.76–0.96)	0.76				
Shrubland	7	0.94(0.82–1.07)	0.50				
Deciduous Forest	7	1.08(0.95–1.22)	0.50				
Spatial dependence							
1 km	0			λ = N/A			
5 km	344			λ = 0.00			0.962
10 km	519			λ = 0.03			0.171
15 km	565			λ = 0.02			0.256
20 km	580			λ = 0.01			0.157
109.7 km	600			λ = −0.01			0.352

^*^p-values from the Likelihood Ratio Test. Spatial correlation parameter (λ). ^a^ VJDHSP = USDA Voluntary Johne’s Disease Herd Status Program for Cattle program

According to the published literature, herd size, testing frequency, and geographical region are associated with JD [21, 29]. Information on those three variables was extracted from the MNDHIA database. Data on herd size and herd turnover rates (454/600 herds) were available in the form of snapshots at the beginning of 2014, 2015, 2016, and 2017. Herd turnover rate calculations were performed by MNDHIA, based on the recommendations by Fetrow et al. [45]. Both herd sizes and herd turnover rates were averaged across the years for this cross-sectional analysis. Farm addresses were verified and geocoded using ArcMap version 10.3.4 [46]. Because the spatial dependence of JD risk has been described for neighboring farms [44], the possible existence of a spatial pattern in the risk of JD for neighboring farms located within 5 through 20 km was accounted for in the analyses.

The voluntary participation in JD control programs has also been described as a factor associated with JD status in a farm [30, 47]. In the absence of data from a control program, information on whether farms included in this study were currently participating in the Voluntary Johne’s Disease Herd Status Program for Cattle program (VJDSHP) was retrieved from the Minnesota Board of Animal Health [9, 29]. VJDSHP was introduced by USDA APHIS in 2002 as a gradual process which allows to recognize herds with low JD prevalence or free from the disease [48, 49]. As of 2017, among the study population, 24/600 (4%) herds were part of the Voluntary Johne’s Disease Herd Status Program (VJDHSP).

Layers of soil pH and soil type in Minnesota were obtained from the Natural Resource Conservation Services of the United States Department of Agriculture (USDA) [25, 50, 51]. The hydrologic soils data were used to estimate the runoff potential of the soils, using the Hydrologic Soil map available from the Web Soil Survey [52]. Because of the scarcity of accurate data, soil iron content was not considered in the study. Information on agroecological biome features such as grassland, shrubland, forest, and cropland were obtained from the Minnesota Geospatial Commons (https://gisdata.mn.gov/), which was based on remote sensing data creating the Land Cover Data Portal under the National Gap Analysis Project (GAP) [53]. The Minnesota GAP classification level 2 land-use/land-cover class code of the GAP data layer was used in the analysis (Additional file 2: Table S1).

### Data analysis

#### Representativeness of MNDHIA herds

Minnesota counties were classified into terciles based on the number of NASS dairy herds present (1 to 12, 13 to 42, and > 43 dairy herds per county). Counties without records for milk cow herds in the NASS 2012 statistics were excluded for this calculation (*n* = 1; Cook county). To evaluate whether the study herds were representative of all Minnesota dairy herds, the number of study herds per county (tested for JD) was compared to the appropriate sample size, i.e. number of dairy herds to be sampled from each county to estimate the true herd prevalence of JD using an imperfect test and adjusting for a finite population (calculated using the AusVet EpiTool Epidemiological calculator (URL: http://epitools.ausvet.com.au) [54, 55]. Total number of herds present in each county was extracted from the National Agricultural Statistical Services (NASS) 2012 Census of dairy herds [18]. In addition, sample size calculations assumed an expected true herd prevalence of JD of 60% [13], a desired herd-level sensitivity of 70% and herd-level specificity of 70% [56], a precision for the estimate of +/− 10% and a level of confidence of 80%. The number of herds included in the MNDHIA database was then compared to the sample size required to accurately estimate prevalence.

#### Descriptive statistics and spatial pattern recognition

The apparent JD prevalence, the spatial distribution of JD milk ELISA test-positive farms, and the presence of spatial autocorrelation in the risk of JD and in other covariates considered were visualized and, for the latter, estimated using Morans’ I and Getis Ord Gi* statistics [19, 20, 57]. Morans’ I statistics measures the overall spatial autocorrelation of the herds based on both locations and value of the variable simultaneously [57]. The Getis Ord Gi* recognizes areas where the local sum of values for a given variable significantly differ from the expected location sum [19, 20]. This statistic identifies herds with high value of the variable next to herds with high values of the variable (high-high clusters), herds with low value of the variable next to herds with low values of the variable (low-low clusters), and no-matching pairs (high-low, or low-high values). Categorical variables, not suited for Getis Ord Gi* analysis, were mapped for visualization (Fig. 2).

#### Classification of JD status

The output variable (herd JD status) was dichotomized as follows: 1) ‘Negative herds’ in which an appropriate number of unique cows were tested during the study period and were all negative (see below definition of appropriate number) and 2) ‘Positive herds’ in which at least one cow was tested positive on the milk ELISA test during the entire study period. The appropriate sample size to certify disease freedom in a herd, i.e. minimum required sample size per herd, using an imperfect test and adjusting for a finite population, was set for each herd using the AusVet EpiTool Epidemiological calculator (URL: http://epitools.ausvet.com.au) [54, 55]. Assumptions for the calculations were a) design prevalence, i.e. expected within-herd prevalence when infected, of 10% [58], and b) sensitivity of the diagnostic test of 52% (IDEXX Laboratories Inc., Maine, USA). Herds that tested fewer cows than the appropriate sample size and that had no positive cows were excluded from the analysis because their apparent JD disease freedom/negative state could not be reliably demonstrated.

#### Regression analysis

The outcome variable used in all models was the farm-level JD milk ELISA test-status (positive, negative). The herd size, which ranged between 1 and 1929 cows, was categorized using Jenks natural breaks method [59]. Similarly, herd turnover rate was categorized into three based on Jenks natural breaks. The testing frequency per herd during the 2.5 years varied between once and 30 times, and was categorized into four classes (1, 2 to 5, 6 to 20, and > 20). Soil pH values ranged between 5.6 and 7.5, and were categorized into three (< 6.0, 6.0 to 7.0, and > 7.0) groups. The soil type/texture was re-categorized into four classes based on the percentage of different types of particles as clay (> 50% clay and ≤ 50% silt), sand (> 50% sand and ≤ 50% clay), silt (> 50% silt and ≤ 50% sand), and loam (equal proportions of sand, silt and clay, i.e. ≤50% sand, ≤50% clay, and ≤ 50% silt), following the soil texture triangle model described elsewhere [60, 61]. Soil hydrologic characteristics were summarized into four categories: 1) Type A with low runoff potential, 2) Type B with moderately low runoff potential, 3) Type C with moderately high runoff potential, and 4) Type D with high runoff potential, when completely wet [51, 52, 61]. Agroecological biome features included four categories, namely, crop/grassland, non-vegetated land, shrubland, and deciduous forest (Additional file 2: Table S1). The VJDHSP participation status was incorporated into the model as a dichotomous variable (current participant vs. non-participant herds).

To avoid multicollinearity, a simple logistic regression was used to assess the marginal association between JD status and the covariates. The strength of the association between pairs of covariates was analyzed using Chi-square test followed by a Cramer’s V test. Variables with *p*-value < 0.2 and that were not significantly associated among them (i.e., Chi-square *p*-values > 0.05 and Cramer’s V > 0.5) were tested as candidate variables in the full multivariable logistic regression model. To prevent overfitting, the full model, including all possible 2-way interactions deemed biologically plausible, was subjected to backward stepwise regression based on the lowest Akaike Information Criterion (AIC) until the most parsimonious (final) model was fitted. The regression analysis was carried out using the R Statistical Software (Foundation for Statistical Computing, Vienna, Austria). For the descriptive analysis and logistic regression, we used R packages ‘Base’ [62] and ‘MASS’ [63].

Evidence of spatial autocorrelation in the residuals of the final regression model was assessed using the global Morans I and local Getis Ord Gi* tests. To account for potential spatial dependence in the outcome variable, a proper conditional autoregressive model (CAR) structure was included in the model [64] using the “spdep” R package [65, 66]. Distances of 1 km, 5 km, 10Km, 15 km, 20 km and the minimum distance between herds that guaranteed all herds had at least one neighbor were tested alternatively to define the neighborhood matrix in the CAR [67]. The isolated herds without a neighbor at each distance thresholds were assigned a lag value of zero at each model fit. The AIC values and significance of the spatial correlation parameter of the CAR model (λ) from the regression models with and without the CAR model structure were compared to select the best model [67].

## Supplementary information

**Additional file 1:** **Figure S1.** The study population (*n* = 600; 13% of the 4746 dairy herds in Minnesota [18] compared to the participants in the Minnesota Dairy Herd Improvement Association (MNHIA; *n* = 1741). The 600 herds here 18.7% of the licensed dairy herds in Minnesota with permits to ship milk for human consumption [17]. Between November 2014 and April 2017, there were 723 herds tested for JD at MNDHIA. Among those, 123 were excluded due to locations outside Minnesota (MN) including Iowa (IA), Wisconsin (WI), and South Dakota (SD); herds without location information; and, 3) inadequate sampling.

**Additional file 2:** **Table S1.** Environmental factors assessed for association with Johne’s disease status in Minnesota dairy herds participating in the voluntary testing program conducted by Minnesota Dairy Herd Improvement Association. **Table S2.** Akike information criterion (AIC) values for the model fits.

## Data Availability

The datasets used during the current study is property of Minnesota Dairy Herd Improvement Association and may be available from the corresponding author on reasonable request and DHIA approvals.

## Abbreviations

CAR: Conditional autoregressive model
ELISA: Enzyme-Linked Immunosorbent Assay
GAP: National Gap Analysis Project
JD: Johne’s disease
MAP: *Mycobacterium avium subsp. paratuberculosis*
MNDHIA: Minnesota Dairy Herd Improvement Association
NASS: National Agricultural Statistics Services
USDA: United States Department of Agriculture
VJDSHP: Voluntary Johne’s Herd Status Program for Cattle program
