# Supplementary figures and images for: Use of a voluntary testing program to study the spatial epidemiology of Johne’s disease affecting dairy herds in Minnesota: a cross sectional study
Source: BMC Vet Res. 2019 Dec 2;15:429. doi: 10.1186/s12917-019-2155-7 (PMC6889654; doi:10.1186/s12917-019-2155-7)

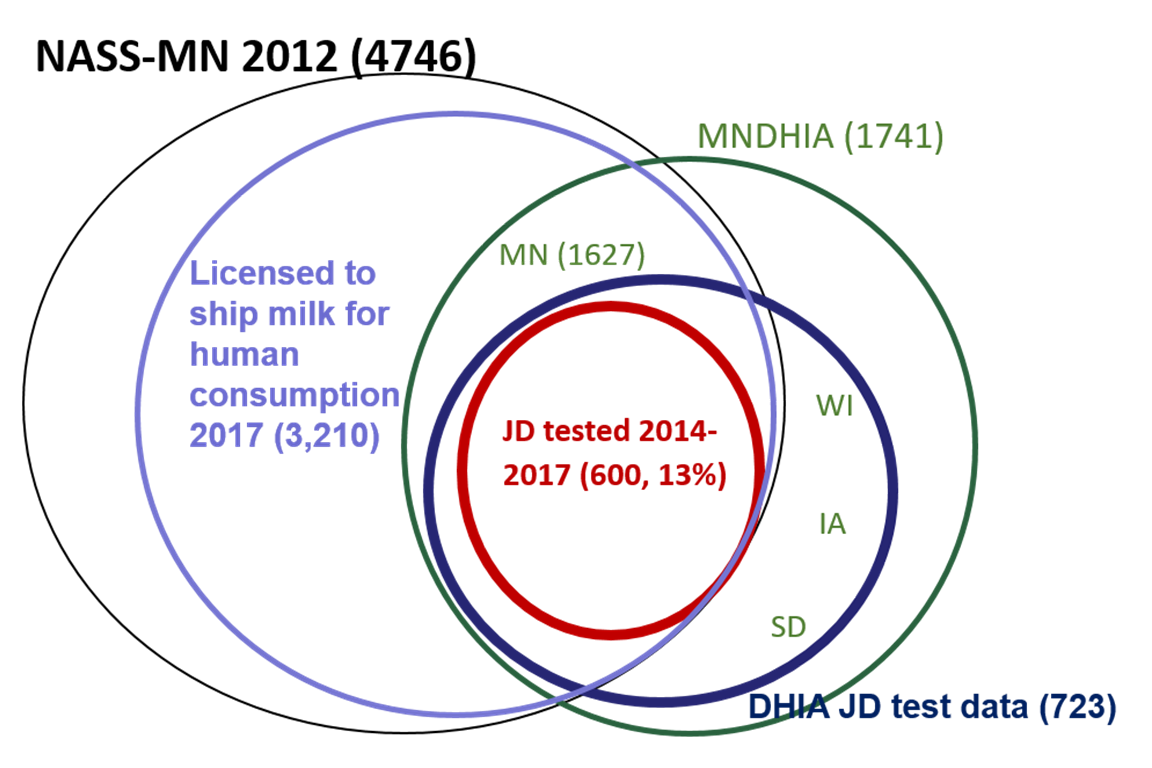

Supplement: Supplementary file 1 — Additional file 1: Figure S1. The study population (n = 600; 13% of the 4746 dairy herds in Minnesota [18] compared to the participants in the Minnesota Dairy Herd Improvement Association (MNHIA; n = 1741). The 600 herds here 18.7% of the licensed dairy herds in Minnesota with permits to ship milk for human consumption [17]. Between November 2014 and April 2017, there were 723 herds tested for JD at MNDHIA. Among those, 123 were excluded due to locations outside Minnesota (MN) including Iowa (IA), Wisconsin (WI), and South Dakota (SD); herds without location information; and, 3) inadequate sampling. [file 12917_2019_2155_MOESM1_ESM.tif]
