# Supplementary material for: Use of a voluntary testing program to study the spatial epidemiology of Johne’s disease affecting dairy herds in Minnesota: a cross sectional study
Source: BMC Vet Res. 2019 Dec 2;15:429. doi: 10.1186/s12917-019-2155-7 (PMC6889654; doi:10.1186/s12917-019-2155-7)
Supplement: Supplementary file 2 — Additional file 2: Table S1. Environmental factors assessed for association with Johne’s disease status in Minnesota dairy herds participating in the voluntary testing program conducted by Minnesota Dairy Herd Improvement Association. Table S2. Akike information criterion (AIC) values for the model fits. [file 12917_2019_2155_MOESM2_ESM.docx]

**Table S1**. Environmental factors assessed for association with Johne’s disease status in Minnesota dairy herds participating in the voluntary testing program conducted by Minnesota Dairy Herd Improvement Association.

| Variable | Description and relevant references | Data source |
| --- | --- | --- |
| 1. Soil type (texture) | Soil types were re-categorized for this study based on the percentage of the clay, sand, and silt content, as seen in the figure below. The solid lines dividing the USDA soil classification i.e. USDA textural triangle arms at 50% were used for the reclassification.  The original figure of USDA soil textural triangle was taken from the publication by Davis and Bennett, 1927 [42] | Gridded Soil Survey Geographic (gSSURGO) Database for the Conterminous United States - 10 meter resolution. (<http://datagateway.nrcs.usda.gov>) [40] |
| 1. Soil pH | Values ranged between 5.6 and 7.5. Missing data were represented with a zero | Gridded Soil Survey Geographic (gSSURGO) Database for the Conterminous United States – 10 meter resolution. (<http://datagateway.nrcs.usda.gov>) [40] |
| 1. Hydrologic soil group (run-off potential) | Seven categories: A, B, C, D, A/D, B/D, and C/D. A/D, B/D, C/D, and D categories were combined to one category and labelled “D” for the analysis here. | Web soil Survey. (<https://websoilsurvey.sc.egov.usda.gov/>) [43] |
| 1. Agroecological zone | The National Gap Analysis Project (GAP) vegetation information were categorized into four levels.  The Level 2 categorization was used in the analysis. | Upper Midwest Gap Analysis Program (UMGAP)(http://www.umesc.usgs.gov/reports_publications/psrs/psr_1999_04.html ) of the U.S. Geological Survey. 30-meter resolution. (gapanalysis.usgs.gov) [44] |

References: [40], [42], [43], and [44]

40. Soil Survey Staff. Gridded Soil Survey Geographic (gSSURGO) Database for State name. United States Department of Agriculture, Natural Resources Conservation Service. Available online at: http://datagateway.nrcs.usda.gov/. 2018. (FY2018 official release). Accessed 10 Feb 2018.

42. Soil survey staff. USDA soil texture triangle classification. Based on: Davis, R. O. E., Bennett, H. H., 1927. Grouping of soils on the basis of mechanical analysis, Dep. Circ. 419, U.S. Dep. of Agric., Washington, D. C. 2017. Available online at: <https://www.nrcs.usda.gov/Internet/FSE_DOCUMENTS/stelprdb1044818.pdf>.

43. Soil Survey Staff. Hydrologic Soil Group. Natural Resources Conservation Service, United States Department of Agriculture. Web Soil Survey. Available online at: [https://websoilsurvey.sc.egov.usda.gov/](https://websoilsurvey.sc.egov.usda.gov/?referrer=Citation.htm-HomeLink1). 2018. Accessed 10 Jan 2018.

44. National Gap Analysis Project. Upper Midwest Gap Analysis Program (UMGAP) of the U.S. Geological Survey. 30-meter resolution (gapanalysis.usgs.gov). Accessed through Minnesota Geospatial commons (Available online at: ftp://ftp.gisdata.mn.gov/pub/gdrs/data/pub/us_mn_state_dnr/biota_landcover_gap/metadata/metadata.html)

**Table S2**. Akike information criterion (AIC) values for the model fits.

| Model | AIC |
| --- | --- |
| Final regression model | 591.47 |
| Final regression model with iCAR at following distances: |  |
| 1Km | 640.64 |
| 5Km | 640.65 |
| 10Km | 638.78 |
| 15Km | 639.36 |
| 20Km | 638.65 |
| 109.7Km | 639.79 |
